# Advancing motor rehabilitation for adults with chronic neurological conditions through increased involvement of kinesiologists: a perspective review

**DOI:** 10.1186/s13102-021-00361-6

**Published:** 2021-10-24

**Authors:** Cameron S. Mang, Sue Peters

**Affiliations:** 1 Faculty of Kinesiology and Health Studies, University of Regina, 3737 Wascana Parkway, Regina, SK S4S 0A2 Canada; 2 School of Physical Therapy, Faculty of Health Sciences, Western University, London, Canada

**Keywords:** Neurorehabilitation, Exercise, Physical activity, Task-oriented, Community-based

## Abstract

Many people with neurological conditions experience challenges with movement. Although rehabilitation is often provided acutely and sub-acutely following the onset of a condition, motor deficits commonly persist in the long-term and are exacerbated by disuse and inactivity. Notably, motor rehabilitation approaches that incorporate exercise and physical activity can support gains in motor function even in the chronic stages of many neurological conditions. However, delivering motor rehabilitation on a long-term basis to people with chronic neurological conditions is a challenge within health care systems, and the onus is often placed on patients to find and pay for services. While neurological motor rehabilitation is largely the domain of physical and occupational therapists, kinesiologists may be able to complement existing care and support delivery of long-term neurological motor rehabilitation, specifically through provision of supported exercise and physical activity programs. In this perspective style review article, we discuss potential contributions of kinesiologists to advancing the field through exercise programming, focusing on community-based interventions that increase physical activity levels. We conclude with recommendations on how kinesiologists’ role might be further optimized towards improving long-term outcomes for people with chronic neurological conditions, considering issues related to professional regulation and models of care.

## Background

An estimated 1 billion people live with neurological conditions, ranging from stroke and traumatic brain injury to neurodegenerative diseases [1]. After the onset of a neurological condition, people often experience challenges with movement that disrupt activities of daily living and decrease quality of life [2]. For sudden-onset neurological conditions like stroke, post-acute rehabilitation programs that address motor deficits are provided in hospital and/or other clinical settings in the initial months after diagnosis [3, 4]. Although such programs are generally beneficial, most individuals experience functional deficits in movement that persist after their completion [3, 5]. Moreover, many people with neurodegenerative conditions, such as multiple sclerosis and Parkinson’s disease, are not admitted to in- or out-patient rehabilitation programs until a later stage of the disease is reached, and thus experience a progressive loss of motor function over time with minimal rehabilitative support [6, 7].

Persistent and progressive losses in motor function commonly propel people with neurological conditions into a cycle of ever-declining health. Motor deficits interfere with performance of activities of daily living [8–10] and participation in physical activity [10, 11]. Correlational studies suggest that inactivity exacerbates functional deficits and promotes deconditioning [12–14]. In this sense, neurological rehabilitation should not be viewed as a temporary undertaking, but rather as a life-long endeavour. Unfortunately, people with complex, chronic health conditions experience many barriers to participating in rehabilitation (e.g., therapist availability, financial constraints) [15–18]. It is proposed here that greater involvement of kinesiologists in motor rehabilitation for people with chronic neurological conditions (i.e., > 6 months post-diagnosis) could provide a means to overcome some current barriers, delay functional decline, and improve long-term motor outcomes.

The primary aim of the article is to initiate conversation on whether long-term motor rehabilitation for adults with chronic neurological conditions might be advanced through greater and more deliberate integration of kinesiologists into the field. We begin with a brief overview of motor rehabilitation for people with chronic neurological conditions, discussing its premise, its relationship to exercise and physical activity, and barriers to participation. Next, we describe the scope of practice of a kinesiologist with consideration of where kinesiologists might fit within the traditional neurological motor rehabilitation team. Finally, we provide recommendations on how kinesiologists might be better utilized to complement existing motor rehabilitation services for people with chronic neurological conditions. We consider limitations of kinesiologists in working in the neurological rehabilitation field and acknowledge alternative approaches to improving access to long-term neurological motor rehabilitation. The discussion is framed within our research and clinical experiences with neurological rehabilitation in Canada and the United States but are relevant to other countries with similar health care resources.

## Motor rehabilitation for people with chronic neurological conditions

### The premise of chronic neurological motor rehabilitation

Most recovery of motor function after sudden neurological damage occurs in the initial 3 to 6 months post-injury during in- and out-patient rehabilitation [3, 19]. While these initial months are a critical time to capitalize on spontaneous recovery processes [20, 21], research indicates that further gains can be made in later stages of recovery, extending to years after onset of the condition [3, 5, 22]. In chronic stages of neurological conditions, motor rehabilitation benefits may be attributed to improved physical conditioning and experience-dependent neuroplasticity, or the rewiring of neural connections in response to experience [23, 24]. Improvements in physical conditioning are stimulated by cardiorespiratory and resistance exercise training, while experience-dependent neuroplasticity is dependent on exercise that incorporates high volumes of repetitive, task-oriented, motor skill practice [23]. Related to both is the phenomenon of learned non-use, which is the tendency to limit use of the more affected extremity despite residual functional capacity in people with hemiparesis [25–27]. With both physical conditioning [28] and experience-dependent neuroplasticity [24] operating on a “use-it-or-lose-it” basis, overcoming patterns of disuse or inactivity after neurological damage are key to improve function or combat further functional declines in all stages of recovery [12–14]. Overall, the documented benefits and activity-dependent nature of physical conditioning and neuroplasticity demonstrate the rationale for, and importance of, providing motor rehabilitation to people with chronic neurological conditions.

### Motor rehabilitation versus exercise programming

Given that chronic neurological motor rehabilitation benefits are partly dependent on physical conditioning, there is a degree of overlap with what might be considered “exercise” or “physical activity” services. A distinction could be made that “rehabilitation” is delivered to improve function or reduce disability associated with a specific underlying condition or impairment [29], while “exercise” or “physical activity” programming focuses on more general health and fitness goals [30]. Yet, the presence of long-term physical disability inherent to chronic neurological conditions blurs the lines. For example, if an individual with chronic hemiparesis participates in a general resistance training program at a community centre, strengthens the limbs on their affected side and experiences improved arm function, we posit that the general resistance training program had a rehabilitative effect. Moreover, the capacity to engage in rehabilitation activities designed to address a specific functional deficit (e.g., muscle weakness) could plausibly be limited by low physical fitness [31] or fatigue [32] that might be addressed by general exercise programming. Another key intersection between the terms is demonstrated by exercise influences on neuroplasticity linked to motor rehabilitation effects [33–35]. Thus, in this article, we operate under the view that when people with chronic neurological conditions engage in exercise or physical activity programming, it can be regarded as a component of motor rehabilitation if the programming affects impairment, activity limitations, or participation restrictions.

### Barriers to chronic neurological motor rehabilitation

Generally, the neurological motor rehabilitation team includes an inter-disciplinary group of physiatrists, physical therapists, and occupational therapists, with supporting contributions from other health care providers such as recreational therapists, psychologists, and nurses [3, 4, 36]. While people with neurological conditions may have regular contact with these professionals during in- and out-patient rehabilitation, access to these services is greatly reduced in the chronic phase [15, 18, 37]. In our experience, some private clinics have specialized therapists who provide therapy on an ongoing basis for clients with chronic neurological conditions; however, generally low availability, high cost of specialized therapists, and limitations on number of funded therapy visits have been described as barriers in past work [16, 17, 37]. Location of services is another barrier to accessing rehabilitation services for people outside of urban centres [17, 38]. With more people experiencing and living longer with neurological conditions [2, 29] and known benefits associated with motor rehabilitation in late stages of neurological recovery, it is critical that new approaches are developed to overcome these barriers and improve access to ongoing motor rehabilitation services for people with neurological conditions.

## Kinesiologists and chronic neurological motor rehabilitation

### What is a “kinesiologist”?

Kinesiology broadly refers to the scientific study of human movement [39]. It is an evolving and multi-disciplinary field that bridges biophysical, sociocultural, psychological, and neuromotor aspects of human movement and performance [40]. In this article, a kinesiologist is considered to be an individual with a minimum of an undergraduate kinesiology degree from an accredited institution. Kinesiology practice is defined as “the assessment of human movement and performance, and its rehabilitation and management to maintain, rehabilitate or enhance movement and performance” [41]. With a wide knowledge base and scope of practice in human movement, kinesiologists can complement many other health care professions [42] and occupy various roles in the exercise, physical activity, rehabilitation, and health industries [43], often providing services at a lower rate than other movement-based practitioners [43].

The broad scope of practice of kinesiologists could be perceived as a strength in terms of versatility of the profession; however, it also contributes to confusion about the distinct role of a kinesiologist relative to other health care professions [43, 44], particularly physical therapists. Important notes are that kinesiologists generally focus on addressing the needs of people with stable health conditions and do not diagnose disorders. Additionally, physical therapists in North America now commonly have graduate degrees and more training in pathology, manual techniques, and various therapeutic modalities than kinesiologists. Although a generalization, it could also be said that many kinesiologists work in community exercise or physical activity centres and most physical therapists in private clinics, home-care, and hospitals [45]. Nevertheless, there remains a need for further discourse to identify the specific role of kinesiologists among other related health care professionals in the field of motor rehabilitation.

### Where do kinesiologists fit in the neurological motor rehabilitation team?

**Fig. 1:**
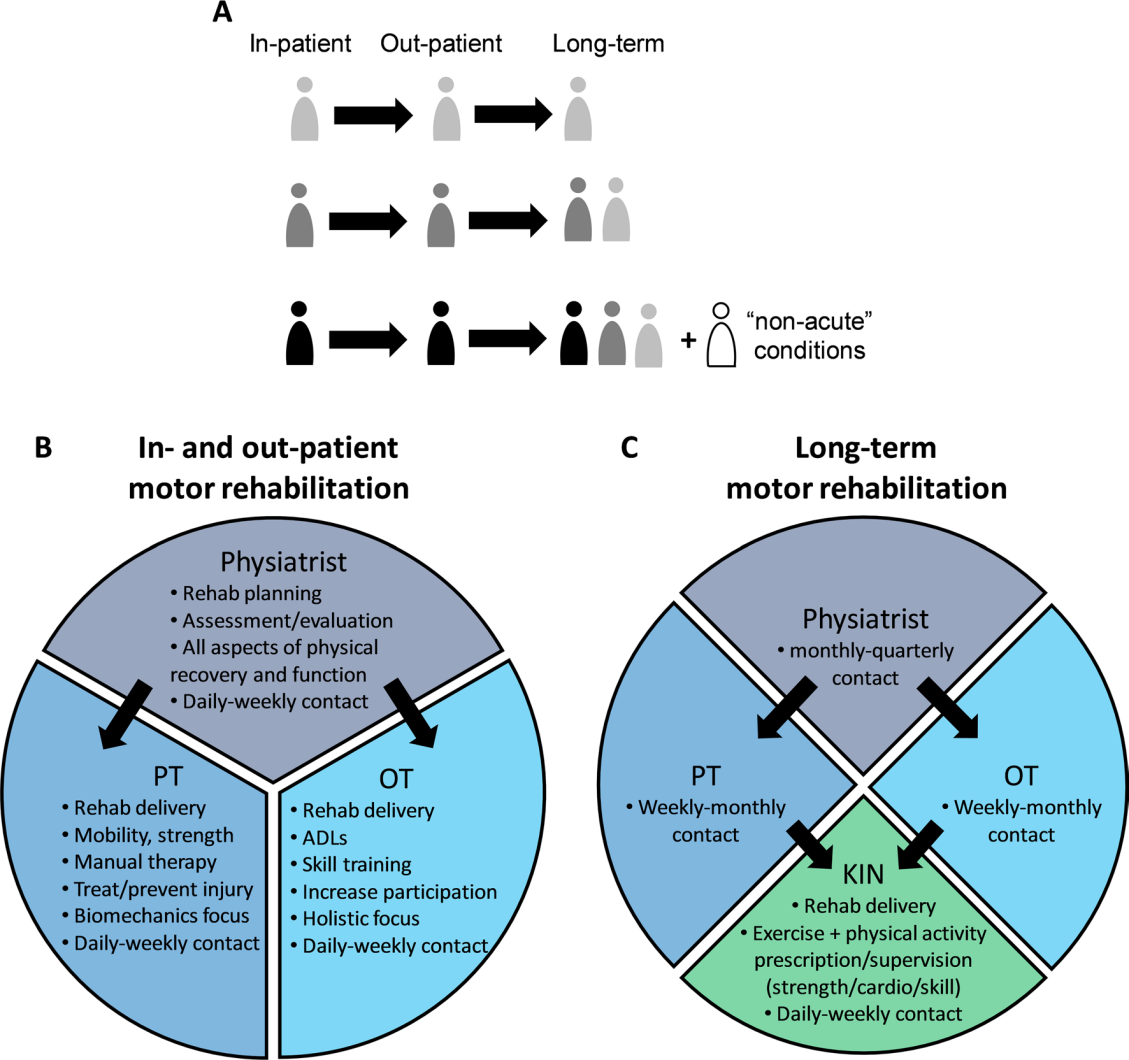
**A** As people with neurological conditions are discharged from in- and out-patient care with persisting motor deficits, and others develop neurological conditions that do not require acute care, the number of people seeking long-term motor rehabilitation supports accumulates. **B** Main health care providers of motor rehabilitation during in- and out-patient rehabilitation. Arrows indicate that physiatrists often refer individuals to physical and occupational therapy (PT/OT). Contact with these health care providers is frequent in these stages. **C** Proposed integration of kinesiologists into long-term motor rehabilitation. PTs and OTs may refer clients to kinesiologists that provide more frequent contact than is typical for physiatrists/PTs/OTs in this stage. Kinesiologists may deliver services at community centres, clinics, or in-home with support from other health care providers. ADLs, activities of daily living

To our knowledge, the practical contributions of kinesiologists to the neurological motor rehabilitation process have traditionally been minimal. With increased evidence that exercise and physical activity benefits most chronic health conditions including neurological conditions [46], there is strong rationale for kinesiologists to be better integrated into the chronic neurological motor rehabilitation team. Moreover, the recognized need for ongoing neurological motor rehabilitation services for people in chronic, stable stages of recovery [5, 15] aligns with the skillset of kinesiologists and the relatively affordable nature of their services [43]. Although the expertise of kinesiologists certainly overlaps with other more traditional members of the neurological motor rehabilitation team, use of their skillset could help to distribute the workload and address the critical need for these services. Figure 1 provides a depiction of the proposed integration of kinesiologists into long-term motor rehabilitation in a role that is complementary and supportive of services delivered by physical and occupational therapists. Although other health care providers are involved in neurological rehabilitation, the figure emphasizes contributions by those most focused on motor aspects of rehabilitation.

A valuable and appropriate contribution that kinesiologists can make through a role in chronic neurological motor rehabilitation is in the delivery of community-based exercise programs. The relatively recent development of community-based exercise programs for people with chronic neurological conditions represents a positive shift in the field towards addressing the need for long-term, affordable motor rehabilitation services. Of note, some existing community-based programs, mainly targeted to people with chronic stroke, already utilize instructors with skillsets consistent with kinesiologist training [47–52]. Here, we highlight two programs developed in Canada: Together in Movement and Exercise (TIME™) [49] and the Fitness and Mobility Exercise (FAME) Program [50].

TIME™ is a community-based group exercise program that serves people who are ambulatory but experience balance and mobility challenges, including people with neurological conditions [49]. In the program, fitness instructors at community centres lead group exercise classes focused on balance and gait. The instructors are certified in cardio-pulmonary resuscitation, first aid, and fitness training (Can-Fit-Pro, 25–30 h certification program) and participate in a training workshop (two half days) led by physical therapists, but do not need to be a kinesiologist (i.e., have an undergraduate kinesiology degree). The Fitness and Mobility Exercise (FAME) program is a similar community-based group fitness class that targets people with chronic stroke who are ambulatory and experience mild to moderate motor impairment [50]. Exercises in the class focus on lower extremity strength, balance, agility, and cardiorespiratory conditioning. Clinical trials involved instructors that were physical therapists, occupational therapists, recreation therapists, kinesiologists, and fitness instructors [53, 54]. Similar to TIME™, FAME guidelines also do not require instructors to have a degree in kinesiology. However, kinesiologists are suited to this role and their involvement could potentially improve fidelity of program delivery and address barriers to community-based exercise participation related to a perceived lack of fitness instructor expertise in serving people with disabilities [52, 55, 56]. In our experience, the added expertise that a kinesiologist with an undergraduate degree can provide relative to a certified fitness instructor in terms of delivering safe and effective exercise programming in clinical populations is substantial, because of the added training in anatomy, physiology, and movement analysis. Thus, these specialized, community-based exercise programs are prime examples of services matched to the skills of a kinesiologist and known to improve long-term outcomes for people with chronic neurological conditions.

Use of kinesiologists in community settings could move beyond general exercise programming to also include programs focused on re-acquisition of motor skills through task-oriented motor skill practice. Researchers in kinesiology-related departments across the world have advanced understanding of the neural control of movement and skill acquisition, and influenced the field of neurological rehabilitation [e.g., 57–61]. Likewise, an essential component of kinesiologist training focuses on motor control, motor learning, and the underlying neural substrates [40], expertise that applies to skill re-learning after neurological damage [24]. While some aspects of balance, agility, and gait training common to exercise programs incorporate task-oriented strategies [49, 53], we are not aware of kinesiologist-led programming with a focus on upper-extremity motor skill training. An example of a program that could be implemented on a larger scale through kinesiologists is the Graded Repetitive Arm Supplementary Program (GRASP), a strengthening, fine motor, and task-oriented upper-extremity motor rehabilitation program developed for people with stroke [62, 63]. With training from therapists like that developed for the FAME program, employing kinesiologists to offer such task-oriented exercise programs in community settings to groups of people with chronic neurological conditions could provide an efficient, affordable, and practical means to promote continued skill-related gains in motor function.

Outside of community-based group program delivery, kinesiologists commonly work alongside physical and occupational therapists in private practice [43]. In clinics specializing in neurological rehabilitation, kinesiologists may support therapists in prescription, monitoring, and progression of exercise and activities like task-oriented skill training, as well as education of clients on healthy lifestyle and physical activity behaviours. In this context, kinesiologists would become increasingly involved in the rehabilitation process as the individual’s neurological condition stabilizes. In this chronic stage of recovery, physical therapists continue to provide guidance to kinesiologists and oversee treatment of any acute or complex clinical issues that arise. With this approach, a clinic’s utilization of kinesiologists could plausibly provide individuals with longer-term access to motor rehabilitation, increase training volumes without drastically increasing client expenses, and improve therapist availability for new clients.

Although on a generally small scale that is inconsistent across locales, the examples we provided demonstrate that kinesiologists and people with related skillsets (e.g., fitness instructors) are already being used to support exercise programming for people with chronic neurological conditions. Given the contributions that kinesiologists can, and to some extent already do, make to chronic neurological motor rehabilitation, development of established roles for kinesiologists could be a constructive step for the field toward enhancing long-term functional outcomes for people with neurological conditions. Specifically, the examples provided indicate that kinesiologists could play a crucial role in the expansion of community care for people with chronic neurological conditions. In this role, kinesiologists could complement the traditional neurological rehabilitation team towards providing increased opportunity for people to participate in motor rehabilitation activities on a long-term basis.

## Recommendations to support kinesiologists’ involvement in chronic neurological motor rehabilitation

### Regulation and specializations

A major challenge for the kinesiology profession in working with clinical populations in general is the lack of regulatory bodies that protect the public [43], such as those that exist for other health care professions (e.g., physical and occupational therapy). While many regional kinesiology associations exist, they primarily serve the interests of the profession. In contrast, regulatory bodies are created by government to oversee a profession in the public interest. Regulation of the kinesiology profession has precedent in Canada; the College of Kinesiologists of Ontario is a regulatory body that oversees kinesiologists in the province and gains its authority from the Kinesiology Act of 2007 [41] and the Regulated Health Professions Act of 1991 [64]. As regulatory bodies for kinesiologists emerge, communication among professional organizations, and educational and research institutions will be critical to ensure alignment between the development of the profession, the curricula that support it, and the research prioritized within the scientific discipline of kinesiology.

Although more widespread regulation of kinesiologists is a critical step for the recognition of the profession, kinesiologists’ formal adoption into the neurological motor rehabilitation team may also depend on the establishment of neurological motor rehabilitation as a sub-field within kinesiology. Many undergraduate kinesiology programs offer concentrations of study, with some common concentrations including content relevant to chronic neurological motor rehabilitation (e.g., adapted physical activity). Likewise, related academic programs in “kinesiotherapy” that focus on using exercise as rehabilitation for people with functional limitations have a rich history in the United States [65]. Development of a concentration of study that unifies the elements of these areas that are pertinent to neurological motor rehabilitation could support the capacity of future kinesiologists to contribute to meeting the growing public need for such services. Aligning such a concentration of study with a form of licensure and requirements for continuing education would further enhance credibility. If executed with a unified approach across organizations and institutions, such an initiative would provide opportunity to advance new ideas and better carve out the role of kinesiologists relative to other health care providers.

Given the long-term nature of efforts to create regulatory bodies and establish concentrations of study, it is our current recommendation that kinesiologists working within a neurological motor rehabilitation team should have, at a minimum, an undergraduate degree in kinesiology from an accredited institution and be registered with a local professional association that requires participation in a professional liability insurance program (e.g., Alberta Kinesiology Association [66]). With this approach, kinesiologists do not require direct supervision from other health care providers and are capable of taking responsibility for their own professional services rather than depending on other professionals, such as physicians or physical therapists, to undertake additional liability. Completion and development of specialized training in neurological rehabilitation are also considered beneficial, although there are currently limited options for kinesiologists. Some neurological rehabilitation certifications for allied health professionals exist through universities and professional organizations (e.g., American Physical Therapy Association, 2018 [67]; Brunel University London, 2019 [68]) and an accreditation course for fitness instructors to work with people with stroke was developed in the United Kingdom [69], but most training opportunities are targeted to physical and occupational therapists. Many certifications relevant to kinesiologists are offered by the Canadian Society for Exercise Physiology and American College of Sports Medicine, applying to personal training, group exercise instruction, clinical exercise physiology, inclusive fitness, and cancer exercise training, among others. Development of a neurological motor rehabilitation certification tailored to the kinesiologist scope of practice could be an actionable, short-term initiative to support kinesiologists in working in this area. Such a certification could provide more in-depth education on the pathology of neurological conditions, common neurological symptoms, task-oriented motor skill training, and the kinesiologist role relative to other health care providers. Opportunity to complete such training would potentially increase self-efficacy of kinesiologists for engaging with this population and enhance their skillsets and credibility to do so.

### Models of care

Ultimately, the need for ongoing and often life-long exercise and motor rehabilitation services for people with neurological conditions [3, 5] suggests that clinic-to-community models of neurological motor rehabilitation are needed. Such models involve the establishment of direct relationships between health and community organizations to provide continuing supports to people experiencing long-term health conditions, such as cancer [70] or cardiovascular disease [71]. If applying such a model to neurological motor rehabilitation, kinesiologists are an ideal fit for delivery of the community-based component through programs like TIME™ and FAME. However, one limitation of such standardized programs relates to eligibility requirements that exclude individuals with more severe impairments who may be unable to participate in group classes and require more individualized exercises [72]. With referral from a physician or therapist, another approach might have a kinesiologist devise an individualized exercise program that a person with a more severe chronic neurological condition completes with the kinesiologist’s ongoing support at a community centre [52, 72]. Based on experience overseeing programs delivered in similar individualized formats [73], this approach has several important differences from more standardized programs: (1) greater responsibility is placed on the kinesiologist to prescribe activities, (2) volunteers or caregivers may be required to assist, and (3) the cost of the programming is increased if direct supervision is required. Nevertheless, long-term goals of individuals with diverse needs and abilities can be met. A key consideration is that a clinic-to-community model of care will need to go beyond simply using kinesiologists for community exercise program delivery; a formal connection between the clinic- and community-based practitioners must also be established. Exciting work towards developing partnerships between health care and recreation organizations for delivery of the TIME™ program in Canada is underway [74]. With such partnerships, kinesiologists would not be expected to work independently, but rather to join the neurological rehabilitation team and provide complementary care.

**Fig. 2:**
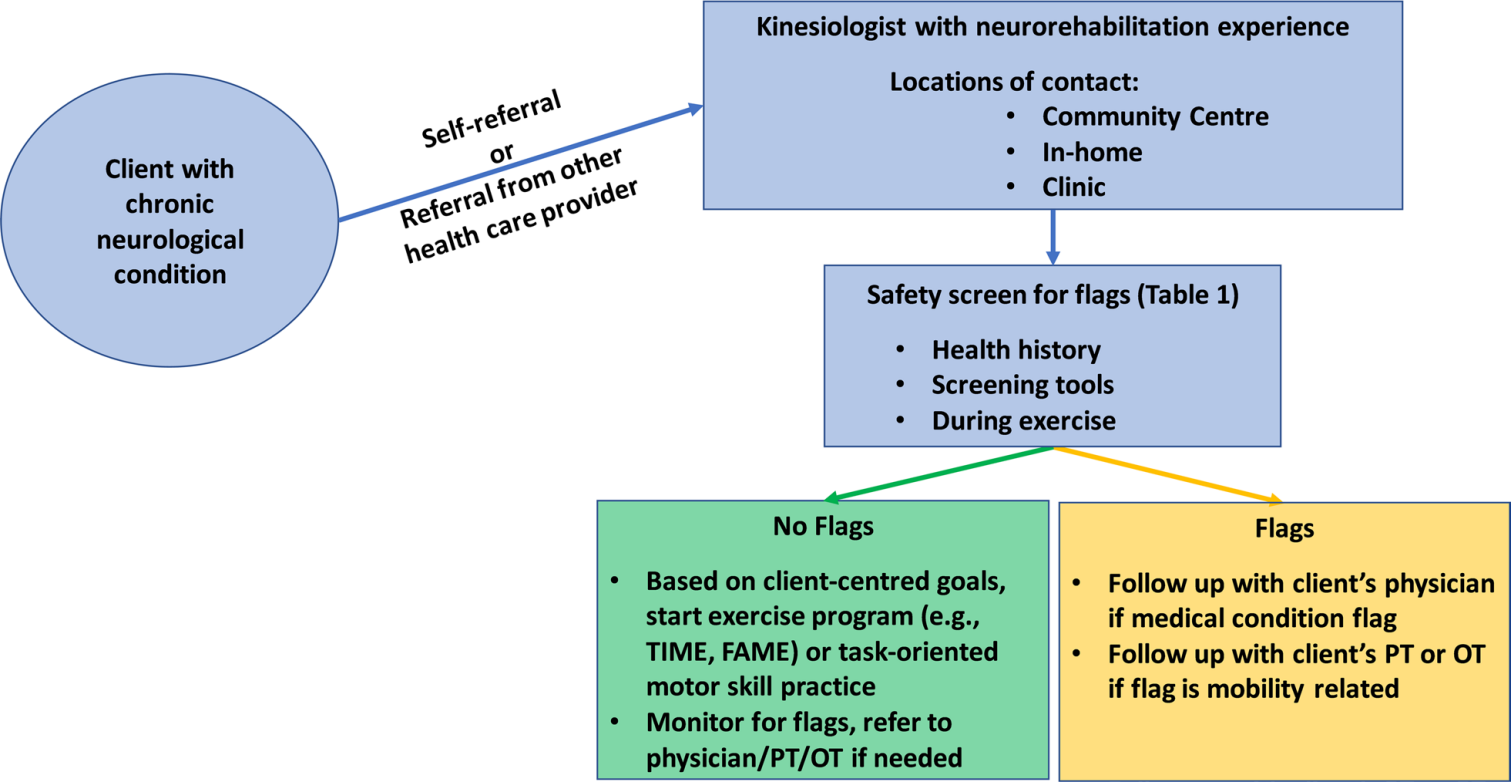
Potential mechanism for integration of kinesiologists into long-term motor rehabilitation for individuals with chronic neurological conditions

In advance of this formal model of care, Fig. 2 depicts an initial mechanism that may support increased involvement of kinesiologists to deliver exercise and motor rehabilitation services to people with chronic neurological conditions. The proposed mechanism allows individuals with chronic neurological conditions to self-refer or to be referred by another health care provider to a kinesiologist working in community centres, inter-disciplinary clinics, or home care. At the first visit, a safety screen for flags could be completed, which may involve one or more of: (1) a thorough health history, (2) completion of standardized screening questionnaires, and (3) further screening or monitoring during each session (Table 1). The safety screen can be used to determine whether clearance from a physician or physical/occupational therapist is needed prior to starting an exercise program with a kinesiologist. Table 1 also provides a non-comprehensive list of common conditions experienced by neurological populations, and symptoms that may require physician follow-up. The setting in which an individual accesses kinesiologist services would depend on client needs that may evolve over time. Community centre programs may be most suitable for clients with lesser impairment. Clinic-based services may provide an extension of out-patient motor rehabilitation for people with varying levels of impairment. Finally, in-home kinesiologist services may be most appropriate for individuals with high levels of impairment and transportation barriers. In all cases, the kinesiologist’s role would be to provide regular, continuing, long-term motor rehabilitation support in consultation with other members of the neurological motor rehabilitation team (refer back to Fig. 1). Although such consultation may be most straightforward in an inter-disciplinary clinic setting, we would encourage such communication across all settings of motor rehabilitation service delivery.

**Table 1:** Safety screening for kinesiologists working with people with chronic neurological conditions

Health History Safety Screen* (not an exhaustive list)	
*Questionnaires*	
CSEP Get Active Questionnaire or equivalent screening tool	
Medical Concerns*	
Recent surgery	Uncontrolled medical conditions
Sudden change in health status	Uncontrolled blood pressure or heart rate/rhythm
Not taking prescribed medications	Uncontrolled heart condition
> 1 chronic health condition	
*Additional screening considerations common in neurological conditions*	
Pain	Pain that limits range of motion may require further assessment
Shoulder subluxation/step deformity	Limit to lower extremity if subluxation present If subluxation corrected, can continue with task-oriented skill practice with caution. Work in pain-free range
Spasticity that limits range of motion	Use caution with task-oriented skill practice
Autonomic dysreflexia	Use caution. Low risk with task-oriented skill practice
Impaired thermoregulation	Use caution as overheating can exacerbate conditions. Low risk with task-oriented skill practice but should be monitored
Altered somatosensation	Use caution with task-oriented skill practice as less somatosensory feedback can lead to injury
Altered sensory processing	Use caution and adapt for sensitivities to light, noise, touch, etc
Altered cognition	May require single step commands during practice
Altered communication	May require adaptations or assistance to communicate
Poor balance	May require individual to hold on during lower extremity task
Poor coordination	May require additional stabilization or guidance
Safety flags during exercise* (not an exhaustive list)	
Angina (chest pain/tightness)	Facial expressions that signify distress
Shortness of Breath or Dyspnea	Significant pain
Excessive sweating (diaphoresis)	Pale, or blueish/greyish appearance (pallor or cyanosis)
Nausea	Light-headedness or confusion
Heart palpitations Numbness/tingling	Exercise related musculoskeletal injury like repetitive strain (risk is low with task-oriented skill practice)
Other unmanaged health concerns	Blurred vision

The listed concerns require follow up with physician. See ACSM’s Guidelines for Exercise Testing and Prescription, 11th ed. [30] for other contraindications to exercise

### Tele-rehabilitation considerations

The use of remote technologies to deliver rehabilitation services could provide an efficient means of delivering in-home motor rehabilitation services to people with mobility and transportation barriers, or those who are geographically isolated from direct services [75]. Beyond accommodation of public health restrictions, physical distancing measures taken due to the COVID-19 pandemic resulted in an accelerated interest in, and broad implementation of, tele-rehabilitation approaches [76–78]. Plausibly, tele-rehabilitation for people with chronic neurological conditions could involve elements of exercise [79] or task-oriented motor skill training [80]. Kinesiologists have the capacity to contribute in each of these areas, possibly by supervising regular tele-rehabilitation sessions and consulting with other professionals. Integration of kinesiologists into tele-rehabilitation delivery could support the potentially high demand under current circumstances and promote the continued utilization of kinesiologists in the future. A separate concern related to the COVID-19 pandemic is that people with neurological conditions, such as stroke, may currently be receiving atypical in- and out-patient rehabilitation experiences such as reduced therapeutic intensity or length of stay [81], potentially leading to greater than usual levels of impairment at discharge. As a result, the need for quality community services for people with neurological conditions will only become more critical. Preparation of kinesiologists to serve people with neurological conditions through tele-rehabilitation could help fill these gaps.

### Alternative approaches

It could be argued that long-term neurological motor rehabilitation needs would be better met by expanding access to regulated health professionals, such as physical and occupational therapists, than by increasing the use of kinesiologists. The more advanced training of these therapists is critical for management of complex clinical issues and use of various relevant treatment techniques and modalities. Potential approaches to expanding access to physical and occupational therapists could involve increasing educational funding, development of programs that do not repeat training acquired by prior kinesiology program graduates, or modification of restrictive reimbursement patterns for therapeutic services. In our view, these are also necessary steps for the field of neurological motor rehabilitation but should not be considered exclusive alternatives to advancing the kinesiologist role. Instead, our view is that a more established role for kinesiologists that respects their proficiencies and limitations in working with this population will support increased access to, and more efficient use of, therapists’ skills and expertise.

## Conclusions

Long-term exercise and physical activity supports for motor rehabilitation for people with chronic neurological conditions are needed. Kinesiologists are movement practitioners with a skillset well-suited to contribute to such strategies, particularly through community-based exercise and physical activity programming. These programs supported by kinesiologists and targeted to people with neurological conditions benefit motor function but are not yet widely offered. Development of relationships among the traditional neurological rehabilitation team and kinesiologists through clinic-to-community models of care could be a step toward advancing kinesiologists’ role in improving long-term outcomes of people with chronic neurological conditions. Yet, lack of professional regulation in most jurisdictions remains a key challenge. Overall, this viewpoint is meant to serve as a starting point for further discussion as we work towards improving long-term motor rehabilitation outcomes for individuals with chronic neurological conditions.

## Data Availability

Not applicable.

## Abbreviations

TIME™: Together in movement and exercise
FAME: Fitness and mobility exercise
GRASP: Graded repetitive arm supplementary program
